# Revealing the molecular mechanisms underlying Xuebijing against sepsis and septic acute kidney injury via bioinformatics and experimental approaches

**DOI:** 10.1371/journal.pone.0333478

**Published:** 2025-10-03

**Authors:** Xing Li, Kaiqi Ren, Michal R. Baran, Juan Tang, Le Wang, Gabriel Mbuta Tchivelekete, Yi Guo, Yang Bai, Weijian Yan, Xinhua Shu

**Affiliations:** 1 Center for Biomedical Innovation and Technology, PuAi Medical School, Shaoyang University, Shaoyang, China; 2 Department of Nephrology, Shaoyang University Affiliated Second Hospital, Shaoyang, Hunan, China; 3 Department of Biological and Biomedical Sciences, Glasgow Caledonian University, Glasgow, United Kingdom; 4 Department of Marine Biology, Faculty of Natural Science, University of Namibe, Moçâmedes, Angola; Northwest Institute of Plateau Biology Chinese Academy of Sciences, CHINA

## Abstract

**Background:**

Sepsis and its related complication acute kidney injury (septic-AKI) are associated with high mortality and morbidity, and have become a global health challenge. Xuebijing (XBJ) injection prepared from five traditional Chinese medicines (TCM) is commonly used for clinical treatment of sepsis and septic-AKI. Yet, the underlying therapeutic mechanism of XBJ is remain elusive. This study aims to unveil the underlying mechanisms of XBJ in treating sepsis and septic-AKI.

**Methods:**

In this study, we used network pharmacology and molecular docking to screen the core drug-disease targets and predict the potential mechanism involving in XBJ against sepsis and septic AKI. Furthermore, *in vitro* experiments were performed to verify the predicted results and clarify the underlying mechanism.

**Results:**

Five hub targets including MMP9, TNF, IL-6, STAT3 and TP53 were identified by constructing and analyzing protein-protein interaction network. Eight key active components linking to five hub targets were also reversely screened. The results of gene ontology (GO) and pathway enrichment analysis showed that on the list of top 10 significant GO terms and pathways, most were inflammatory signaling pathways. The molecular docking results suggested that eight active components more preferentially bound to MMP9 and TNFα with the highest affinity. *In vitro*, XBJ significantly decreased the mRNA and protein levels of IL-1β, IL-6, TNFα and MMP9 in HEK-293 cells exposed to lipopolysaccharides (LPS).

**Conclusions:**

XBJ exerted therapeutic effects on sepsis and septic-AKI through suppression IL-1β/MMP9, IL-6/MMP9 and TNFα/MMP9 at both mRNA and protein level. This study provides a pharmacological basis for further validating the therapeutic mechanism of XBJ in treating sepsis and septic-AKI by *in vitro* and *in vivo* experiments.

## 1. Introduction

According to the Third International Consensus (Sepsis-3) published in 2016, sepsis is currently defined as a life-threatening organ dysfunction caused by a dysregulated host response against infection, while septic shock describes a subset of sepsis patients accompanied with circulatory and cellular/metabolic dysfunction [1]. Sepsis may turn into septic shock under the condition of delay diagnosis and/or without timely appropriate treatment, leading to multiple organ failure or even death [2,3]. One of the most common complications of sepsis is acute kidney injury (AKI), known as septic AKI or sepsis-associated AKI, which is identified as an independent risk factor for mortality, resulting in increased complexity and cost of care. Septic AKI is now recognized as a hallmark of severe sepsis and septic shock, and strongly correlates with prolonged hospital length of stay, unacceptable mortality and morbidity [4]. Sepsis occurs in 45–70% of all cases of AKI among critically ill patients, and has been globally public health concern, bringing about heavy healthcare and economic burdens worldwide [5,6].

Over the past 50 years, the understanding of pathogenesis of sepsis has made great progress. Sepsis is caused by microbial infection, which initiates hyper inflammation and immune response, resulting in release of excess proinflammatory endogenous molecules (especially cytokines) into circulation, known as cytokine storm [7]. Recently, the mechanisms involving in sepsis and septic-AKI including STING-IRF3-NF-κB pathway, complement system, stress signaling mediated via reactive oxygen species (ROS)/NLRP3 inflammasome activation, macrovascular and microvascular dysfunction, cell cycle arrest and apoptosis, metabolic reprogramming and so on, were comprehensively reviewed [6,8]. Based on the advanced understanding of sepsis and septic-AKI, several therapies such as early antibiotics, fluid resuscitation, and hemodynamic support by vasopressors, kidney replacement therapy etc. have been developed [6,9]. Besides the above predominant clinical therapies, therapies derived from complementary and alternative medicines, for instance, traditional Chinese medicine (TCM) formulae, like Xuebijing injection (XBJ), Shengmai injection, Huanglian Jiedu decoction, Dachengqi decoction, etc., have also been applied to clinical practice for treating sepsis and related multi-organ injury [10].

XBJ is an intravenous formulation, which has been widely used for treating critical ill diseases such as sepsis, septic shock, multiple organ dysfunction syndrome, chronic obstructive pulmonary disease [11,12]. XBJ was approved as a State Category II New Drug for treating sepsis in China as early as 2004 (China Food and Drug Administration, No Z20040033). This formulation contains five traditional Chinese herbs, including Honghua (*Carthamus tinctorius* L. Safflower, *Asteraceae*), Chishao (*Paeonia lactiflora* Pall. Radix, *Ranunculaceae*), Chuanxiong (*Ligusticum* chuanxiong Hort. Rhizoma, *Umbelliferae*), Danshen (*Salvia miltiorrhiza* Bge. Radix, *Labiatae*) and Danggui (*Angelica sinensis* Radix, *Umbelliferae*). There are nearly 30 bioactive constituents contributing to pharmacological effects of XBJ. Among these ingredients, hydroxysafflor yellow A, paeoniflorin, oxypaeoniflorin, albiflorin, senkyunolide I, and tanshinol are the principal active compounds jointly responsible for anti-sepsis effect of XBJ [12]. The obtained clinical benefits from XBJ are associated with immunomodulatory and anti-inflammatory activity, and ability of eliminating bacteria and viruses, degrading toxins and improving blood circulation. Recently, the anti-septic effect has further been consolidated by two large-scale and rigorous clinical trials [4,13]. Yet, understanding of therapeutic mechanism underlying anti-sepsis of XBJ is still very limited owing to multiple bioactive compounds and multiple therapeutic targets.

To address this issue, strategy of integrating network pharmacology, molecular docking and experimental validation was used to clarify the anti-septic mechanism of XBJ. Network pharmacology utilizes drug, compound, TCM herbs, gene, disease-databases and some softwares/tools like Cytoscape to construct a relationship of “drug-targets-pathways-disease”, which is involved in system biology, bioinformatics and pharmacology [14,15]. Molecular docking is an important computer-aided drug design approach, which was firstly developed for analyzing the conformation and orientation of macromolecular targets and small molecules at the binding site. Now, it can perform different tasks during the course of drug discovery, such as hit identification and optimization, prediction of adverse effects, polypharmacology, drug repurposing, and target fishing and profiling [16,17]. The combination of network pharmacology and molecular docking can handle complex relationships between multi-targets and multi-ligands, which is consistent with the holistic and systemic view of TCM for treating complex diseases. *In vitro* experiments can be performed to preliminarily verify the predicted targets and pathways. Thus, we employ this combining strategy to unravel the mechanism of anti-sepsis and septic-AKI of XBJ injection.

## 2. Materials and methods

### 2.1. Screening of active compounds and their targets

Based on two pharmacokinetic parameters oral bioavailability (OB ≥ 30) and drug likeness (DL ≥ 0.18), active constituents and related targets were collected by retrieving five herbs *Carthami Flos* (Honghua), *Paeoniae Rubra* Radix (Chishao), Chuanxiong Rhizoma (Chuanxiong), *Salviae* Radix (Denshen), *Angelicae Sinensis* Radix (Danggui) against TCMSP database (https://old.tcmsp-e.com/tcmsp.php), respectively. Related targets were merged and dereplicated.

### 2.2. Collecting targets of sepsis and septic-AKI

Disease related targets were collected from six databases, including DisGeNET (http://www.disgenet.org), GeneCards (https://www.genecards.org), Therapeutic Target Database (TTD, https://db.idrblab.net/ttd/) PharmGKB (https://www. pharmgkb.org/), Online Mendelian Inheritance in Man (OMIM, https://omim/org), DrugBank (https://www.chemeurope.com/en/encyclopedia/DrugBank.html). “Sepsis” and “acute kidney injury” were used as searching keywords, and the species parameter was set as “*Homo sapiens*”. In addition, targets from GeneCards and DisGeNET were further filtered out by relevance score > 0, score_gda ≥ 0.1, respectively. In order to keep consistency, the obtained targets were converted into standardized protein gene names using the UniProt database. Subsequently, all targets were merged, and the duplicated targets were removed.

### 2.3. Acquiring common targets of drug and disease

The common targets between drug and disease were acquired by intersecting two sets via the online tool InteractiVenn (http://www.interactivenn.net/), which can also visualize the overlapping targets through Venn plot.

### 2.4. Constructing the protein-protein interaction network

The protein-protein interaction (PPI) was extracted from STRING database (https://stringdb.org/) (accessed on 24 June 2023) with active interaction sources of textmining, experiments and databases, minimum required interaction score of 0.70 (high confidence), and organism limited to “*Homo sapiens*”. Then the PPI was constructed and displayed by Cytoscape V3.8.2. Centiscape2.2 plugin was used to conduct network topological analysis for identifying the hub genes and nodes, which had high degree and betweenness.

### 2.5. Gene ontology and pathway enrichment analysis

The gene ontology (GO) and pathway enrichment analysis was conducted by online tools Metascape (http://metascape.org/gp/index.html) and STRING (https://stringdb.org/) (accessed on 24 June 2023), with defined organism “*Homo sapiens*”. The GO analysis included three modules, biological processes (BP), cellular components (CC), molecular functions (MF). The pathway analysis consisted of enrichment in Kyoto encyclopedia of genes and genomes (KEGG), Reactome and WikiPathway databases. The most significant top10 enriched GO terms and pathways were displayed by bar plot, which were performed with package ggplot2 (V3.4.3) under R environment (V4.2.0).

### 2.6. Molecular docking

To investigate the interaction between ligands and hub targets, molecular docking was performed by using AutoDock Tools (ADT) V4.2 software. In brief, the 3D structures of active compounds potentially associated with hub targets were downloaded from PubChem in SDF format, which then were converted into pdb format by PyMOL V 2.5.5 software and processed by ADT, following saving as pdbqt format. The crystal structures of hub targets were extracted from RCSB Protein Data Bank (PDB) in pdb format, with focus on high resolution, completeness, and human origin. Protein structure was further subjected to a series of optimizing steps by PyMOL and ADT, such as removing water, metal ions and original ligand, calculating charge, adding hydrogen, determining grid box parameters, following the generation of pdbqt file. Subsequently, the interaction between the optimized ligands and proteins was assessed by AutoDock Vina under CMD environment in Microsoft OS. The ligand with best docking conformation was merged with processed protein structures for profiling possible interactions by online tool Protein-Ligand Interaction Profiler (PLIP, https://plip-tool.biotec.tu-dresden.de/plip-web/plip/index). Finally, the docking results were visualized with the help of PyMOL and Adobe Illustrator CS6 V16.0.0 softwares. To validate the docking protocol, the root mean square deviation (RMSD) was calculated between the co-crystal ligand and the prepared ligand structure using the align function in PyMOL. The calculated RMSD values were 0.026 Å (TP53, PDB ID: 5O1F), 0.436 Å (TNF, PDB ID: 2AZ5) and 1.814 Å (MMP9, PDB ID: 4WZV), indicating that the docking method was conducted correctly.

### 2.7. Cell viability

A 96-well plate was seeded with human embryonal kidney 293 (HEK-293) cells (purchased from ATCC, http://atcc.org, *Homo sapiens*, CVCL_0045, Cat. No. CRL-1573) at a density of 5 × 10^4^ cells/well and incubated for 24 h. XBJ was purchased from Tianjin Chasesun Pharmaceutical Co. Ltd (Cat. No. Z20040033) and contained 123 compounds (listed in the patent file, CN110632230, https://patents.google.com/patent/CN113791154B/zh) identified by the company with high-pressure liquid chromatography-mass spectrometry (HPLC-MS). The cells were treated with varying concentration of XBJ for 24 h, media were removed and the cells were washed with PBS twice. 50 µl of thiazolyl blue tetrazolium bromide (MTT, 0.4 mg/ml, purchased from Merck Life Science UK Limited, Glasgow, Scotland) was added in each well and incubated for 2 h at room temperature (RT). The MTT reagent was removed and the MTT-formed crystals were dissolved using dimethyl sulfoxide (100 µL per well) on an orbital shaker for 20 min. The absorbance was measured at 570 nm in a microplate spectrophotometer Epoch reader.

### 2.8. Immunocytochemistry

HEK293 cells were seeded in a 12-well plate with coverslip at density of 5 × 10^5^/well and incubate for 24 h. Cells were treated with lipopolysaccharides (LPS, Cat. No. LPS25, purchased from Merck Life Science UK Limited, Glasgow, Scotland; 5 µg/ml based on previous publications), XBJ (5%, based on the above cell viability assay) or LPS + XBJ for 24 h. The media were removed and cells were washed with PBS twice, then fixed with methanol at −20°C for 5 min. The cells were blocked with 2% bovine serum albumin (BSA, Cat No. A7030, purchased from Merck Life Science UK Limited, Glasgow, Scotland) for 30 min at RT, then incubated with anti-MMP9 antibody (Cat. No. 10375–2-AP, 1:200 dilution from Proteintech) for 1 h at RT. After washing with PBS for three times (5 min each time), the cells were incubated with secondary antibody (Cat. No. A11037, 1:500 dilution from Thermo Fisher Scientific) for 1 h at RT. After washing with PBS for five times (5 min each time). The cells were mounted with VECTASHIELD Mounting Medium containing 4’,6-diamidino-2-phenylindole (DAPI). Signals were captured using confocal microscopy (LSM800 with Airyscan, ZEISS). To quantify the signal intensity, CZI files of images obtained using ZEN 3 blue software were imported into ImageJ (https://imagej.net/ij/). Signal intensity of one area (200 µm × 200 µm with Bit Depth: 8 Bit and Pixel scale: 0.050 µm × 0.050 µm) of each image was measured, five images of each group were used for the quantification. Total Mean intensity was obtained and was adjusted to a percentage by mean intensity treatment group/ mean intensity control ×100.

### 2.9. Quantitative real-time reverse transcription polymerase chain reaction (qRT-PCR)

HEK293 cells were seeded in a 12-well plate at density of 5 × 10^5^/well and incubate for 24 h. Cells were treated with LPS (5 µg/ml), XBJ or LPS + XBJ for 24 h. After washing with PBS twice, RNAs were extracted using the TRIzol® reagent (Cat. No. T3934, MERCK) and cDNA was synthesized using SuperScript™ III Reverse Transcriptase (Cat. No. 18080093, Thermo Fisher Scientific) following the manufacturer’s protocols. mRNA levels of IL-β, IL-6, TNFα and MMP9 were detected using Applied Biosystems™ PowerTrack™ SYBR Green Master Mix (Cat. No. 16525231, Fisher Scientific) based on the manufacturer’s guidance. The primers for these genes were listed in S1 Table.

### 2.10. Enzyme-linked immunosorbent assay (ELISA)

Proinflammatory cytokines were detected by ELISA using human IL- 1β, IL-6, and TNFα Mini ABTS ELISA Development Kits (PeproTech, UK) respectively, following the manufacturer’s instruction. Briefly, 100 µL of IL-1β (0.5 µg/mL), IL-6 (1.0 µg/mL) or TNFα (1.0 µg/mL) capture antibodies was immediately added to each well of the ELISA plate and incubated overnight at RT. The unbound antibodies were removed, and the plate was washed 4 times with the washing buffer and then incubated with blocking buffer for 1 h at RT. Standards of individual cytokines (IL-β, 0–1000 pg/mL; IL-6, 0–2 ng/mL; TNFα, 0–3000 pg/mL) and media from untreated HEK293 cells or HEK293 cells treated with 5% XBJ, 5 µg/mL LPS, or 5 µg/mL LPS + 5% XBJ for 24 h were added in the plate and incubated for 2 h at RT. The plate was washed 4 times with the washing buffer. 100 µL of IL-1β and TNFα (both 0.5 μg/mL) as well as IL-6 (0.25 μg/mL) detection antibody were added, followed by incubation for 2 h at RT. The plate was aspirated and washed 4 times, and avidin-horseradish peroxidase (HRP) conjugate (1:2000) was added (100 µL) and incubated for 30 min at RT, followed by washing for 4 times before adding 100 µL of ABTS liquid substrate that was incubated at RT for color development. Absorbance was detected at 405 nm with wavelength correction set at 650 nm using the Epoch Microplate Reader.

### 2.11. Statistical analysis

Data results were displayed as mean ± standard error of the mean (mean ± SEM) and analyzed using Prism GraphPad 10 (La Jolla, CA, USA) by one-way ANOVA with the Bonferroni test. *p* < 0.05 was considered as significance.

## 3. Results

### 3.1. Identification of potential targets

**Fig 1:**
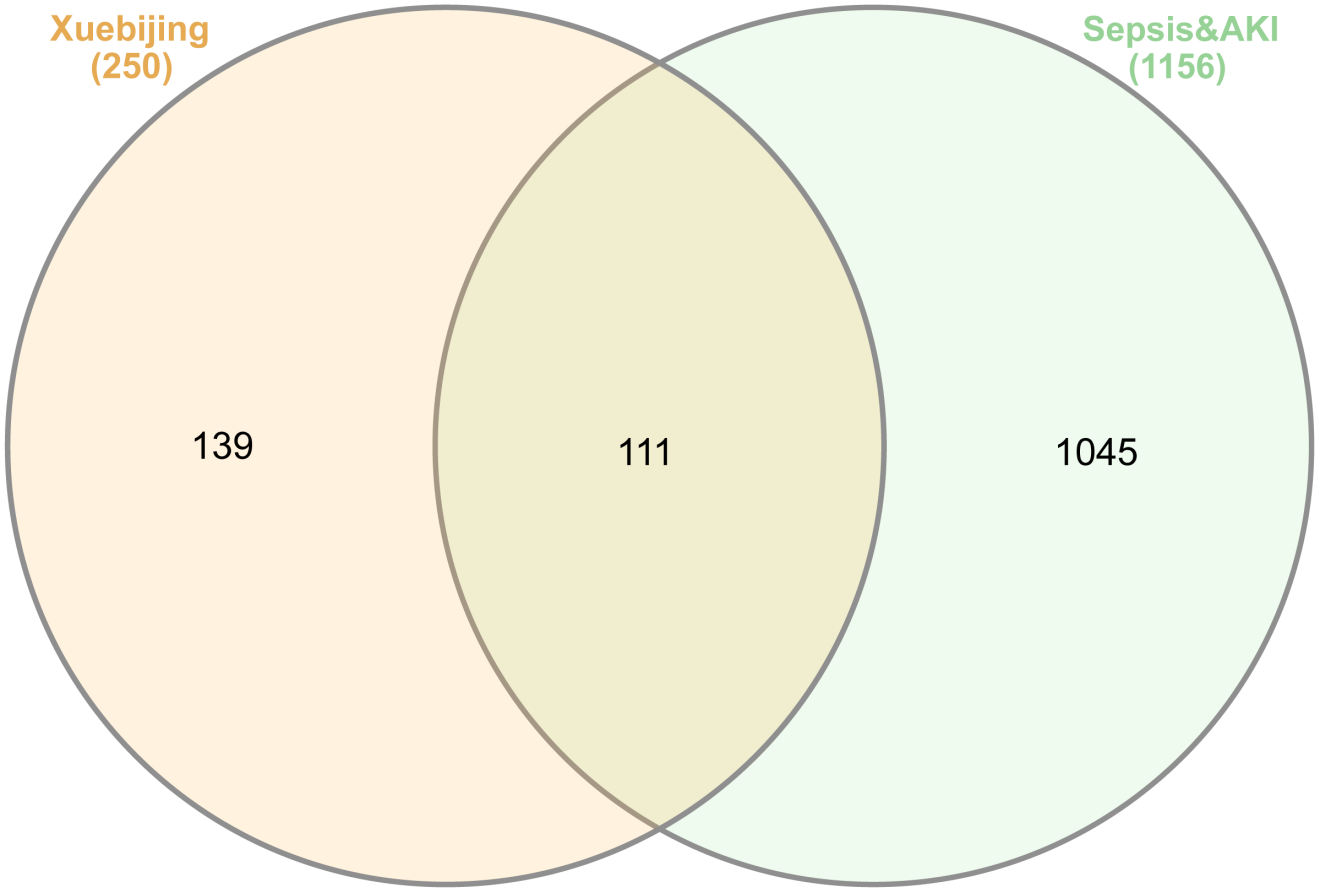
The targets distribution of two sets represented by Xuebijing and sepsis & AKI, respectively. The two sets shared 111 targets. This Venn plot was performed by an online tool InteractiVenn.

Five traditional herbs used to prepare XBJ injection were employed to search against TCMSP database for screening and collecting active compounds and related targets. 115 active ingredients that included most ingredients reported in the patent file (CN110632230, https://patents.google.com/patent/CN113791154B/zh), and 250 dereplicated targets were obtained. 604 sepsis-related targets and 728 acute kidney injury-related targets were extracted from six databases including GeneCards, DisGeNet, Drugbank, PharmGKB, OMIM and TTD. The two sets of disease target were merged, resulting in a total of 1156 dereplicated targets. The intersection of XBJ’s target set and sepsis & AKI’s target set was obtained by InteractiVenn tool, containing 111 common targets (**Fig 1**).

### 3.2. Construction of PPI

**Fig 2:**
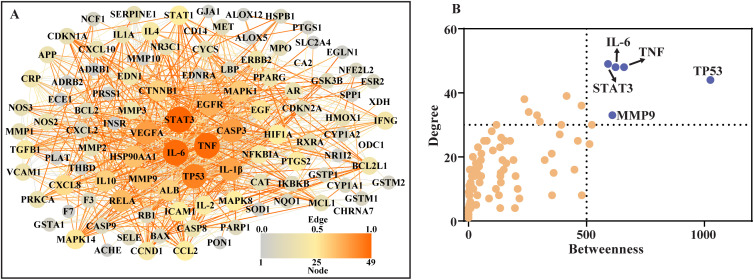
The PPIs network of 111 common targets and identification of hub targets. (A) PPIs network map; (B) Centrality of network nodes displayed by scatter plot. Five hub targets were defined by betweenness ≥ 500 and degree ≥ 30.

**Fig 3:**
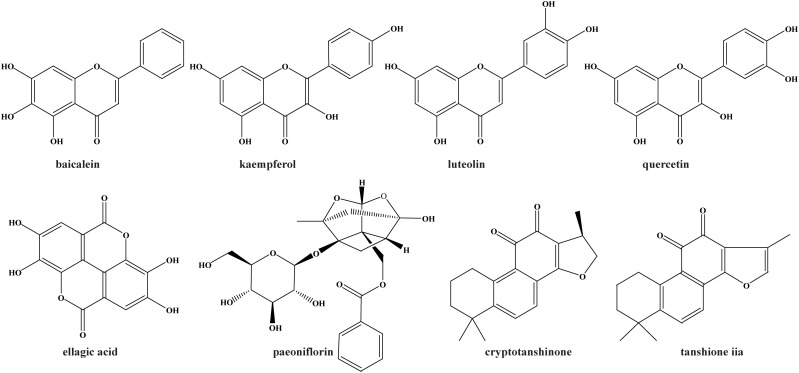
The chemical structure of key active ingredients. The 8 key active components from 5 TCM herbs were screened by the linking of 5 hub targets.

To extract the data of interaction between proteins, 111 common targets were imported into STRING with specified organism “*Homo sapiens*”. Then, the PPI map was constructed with Cytoscape software (**Fig 2A**) and subjected to network nodes centrality analysis with Centiscape 2.2 plugin. Based on the betweenness ≥ 500 and degree ≥ 30, five hub targets including IL-6, TNF, TP53, STAT3 and MMP9 were identified (**Fig 2B**). According to the mapping relationship between active compounds and related targets, 8 active components were identified through reverse screening of 5 hub targets. They were baicalein, luteolin, quercetin, kaempferol, tanshinone_iia, cryptotanshinone, ellagic acid and paeoniflorin, illustrated in **Fig 3**.

### 3.3. Biological function analysis

**Fig 4:**
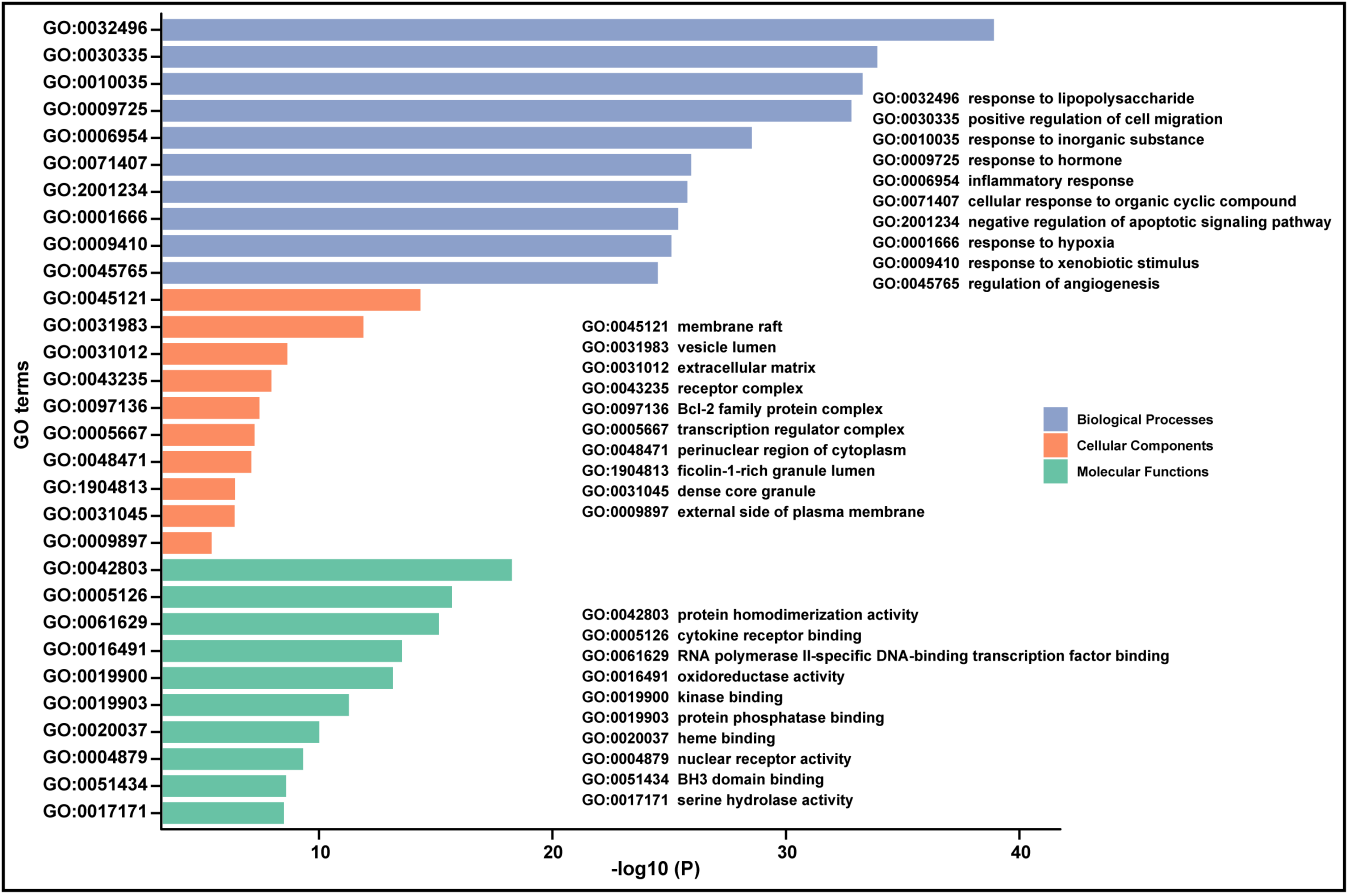
The GO enrichment analysis of 111 common targets by Metascape tool. The GO annotation included biological processes (BP), cellular components (CC), molecular functions (MF).

Gene ontology (GO) is a structured and standardized system for categorizing and describing the functions of genes and their products. In order to further understand the biological function of common targets connecting XBJ injection with sepsis & AKI, 111 overlapping targets were subjected to GO annotations and pathway enrichment analysis. GO analysis by Metascape discovered that common targets possibly acted on several biological processes (BP) including response to lipopolysaccharide (GO:0032496), positive regulation of cell migration (GO:0030335), response to inorganic substance (GO:0010035), response to hormone (GO:0009725), inflammatory response (GO:0006954) and so on (**Fig 4**). The top significant cellular components (CC) annotations mainly encompassed membrane raft (GO:0045121), vesicle lumen (GO:0031983), extracellular matrix (GO:0031012), receptor complex (GO:0043235), Bcl-2 family protein complex (GO:0097136), etc. The top significant molecular function (MF) annotations of common targets comprised protein homodimerization activity (GO:0042803), cytokine receptor binding (GO:0005126), RNA polymerase II-specific DNA-binding transcription factor binding (GO:0061629), oxidoreductase activity (GO:0016491), kinase binding (GO:0019900), and so forth. Moreover, GO annotation was also performed by STRING. The biological processes of response to organic substance (GO:0010033), oxygen-containing compound (GO:1901700), chemical (GO:0042221), stress (GO:0006950) and lipid (GO:0033993) were recognized as top significant GO terms (S1 Fig). Cellular response to chemical stimulus (GO:0070887), organic substance (GO:0071310) and oxygen-containing compound (GO:1901701) were also contained in the top 10 BP annotation list. The CC annotations of common targets by STRING were similar to that of Metascape, encompassing extracellular space (GO:0005615), extracellular region (GO:0005576), membrane raft (GO:0045121), vesicle lumen (GO:0031983), perinuclear region of cytoplasm (GO:0048471). The top 10 MF annotations of common targets were mainly about protein binding, signaling receptor binding, enzyme binding, cytokine receptor binding, protein homodimerization activity.

**Fig 5:**
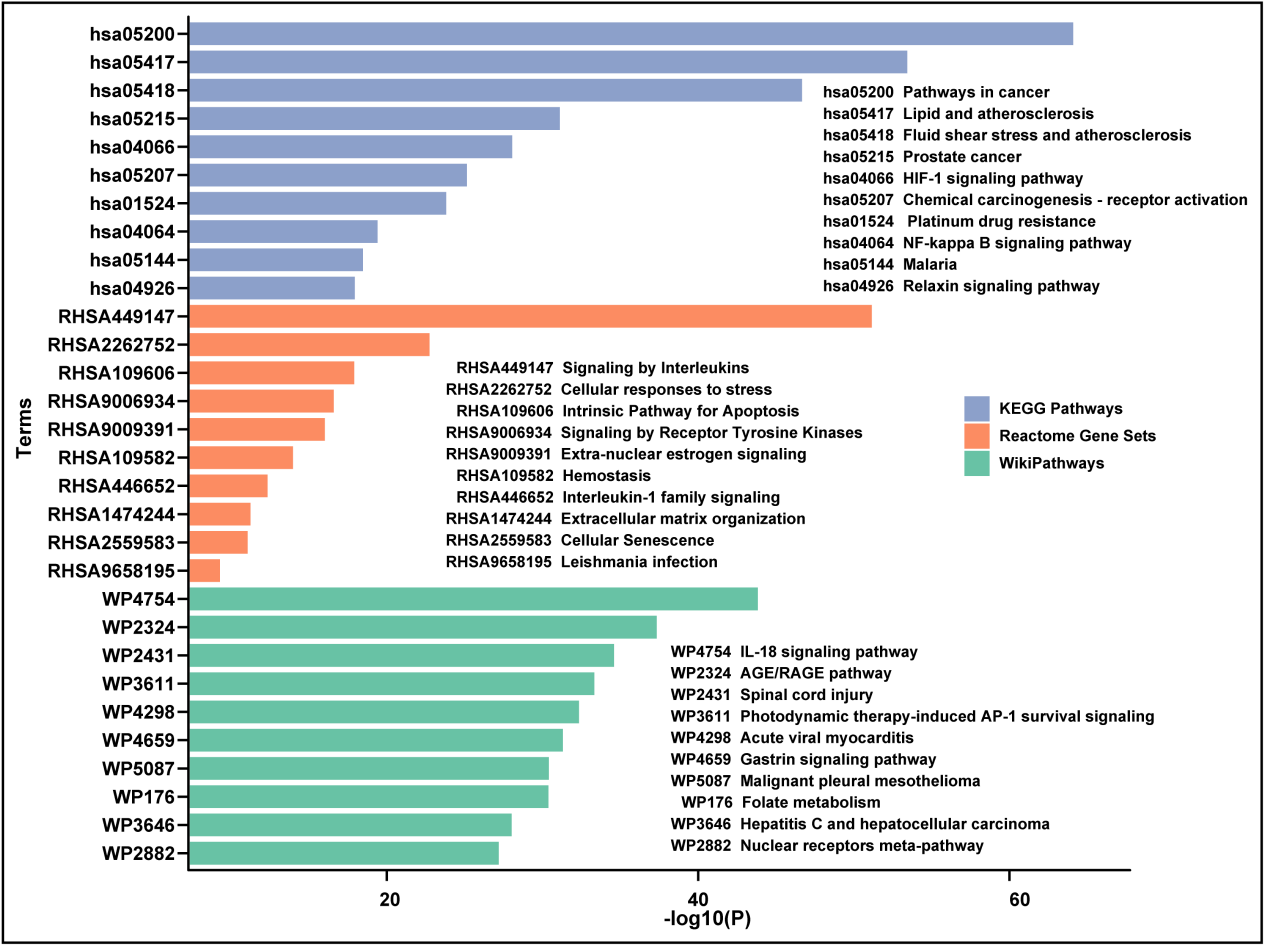
The pathway enrichment analysis of 111 common targets by Metascape tool. The pathway analysis covered three databases, including KEGG, Reactome, WikiPathways.

Pathway enrichment analysis has served as a widely used knowledge-based approach for interpreting and contextualizing biomedical data, facilitating the understanding molecular mechanism of drug treating disease. 111 common targets were employed to pathway analysis with Metascape and STRING, both covering KEGG, Reactome and WikiPathway databases. As the result from Metascape showed that the common targets were involved in HIF-1 signaling pathway (hsa04066), NF kappa B signaling pathway (hsa04064), signaling by interleukins (RHSA449147), cellular response to stress (RHSA2262752), signaling by receptor tyrosine kinase (RHSA9006934), interleukin-1 family signaling (RHSA446652), extracellular matrix organization (RHSA1474244), IL-18 signaling pathway (WP4754), AGE/RAGE pathway (WP2324), which were on the top 10 list of pathways with a *p*-value <0.05 (**Fig 5**). Among the enriched pathways from STRING based on the *p*-value (S2 Fig) and strength (S3 Fig), some were consistent with pathways enriched by Metascape, such as signaling by interleukins, cellular response to stress, IL-18 signaling pathway, AGE/RAGE pathway.

### 3.4. Molecular docking

To unveil the interaction between active components and hub target protein, molecular docking was performed by the ADT tool. For completely assessing the interactions, three sets of grid box parameters and multiple crystal structures were employed to molecular docking. The docking scores (presented as affinity) of eight active ingredients and five hub target proteins (MMP9, STAT3, IL-6, TP53, TNF) were listed in **Table 1**. From the view of targets, luteolin was identified as best ligand of MMP9 (PDB ID: 4WZV), TP53 (PDB ID: 5O1F) and IL-6 (PDB ID: 1ALU), with scores of −11.33, −9.47, −7.20 kcal/mol, respectively (**Table 2**). Cyptotanshinone (CTS) had strongest affinity with STAT3 (−8.82 kcal/mol) and TNF (−9.30 kcal/mol). On the other hand, in the sight of active compounds, MMP9 (PDB ID: 4WZV) was the optimal docking targets of luteolin, quercetin, baicalein, ellagic acid (EA), and tanshinone iia (Tan IIA) with at least the minimum of docking score −9.53 kcal/mol (**Table 3**). In addition, TNF bound with kaempferol (KF), paeoniflorin (PF) and CTS, with optimal affinity (−9.12, −9.09, −9.30 kcal/mol, respectively).

**Table 1:** The docking score of key active components and hub targets (affinity, kcal/mol).

Target	PDB ID	baicalein	KF	luteolin	quercetin	EA	PF	CTS	Tan IIA
MMP9	1GKC	−9.533	--	−9.735	−9.592	−7.387	--	--	−8.097
	1L6J	−9.614	--	−10.050	−9.860	−8.947	--	--	−8.579
	4WZV	−10.500	--	−11.380	−11.140	−9.723	--	--	−9.530
	4XCT	−10.160	--	−10.940	−10.790	−8.180	--	--	−8.816
	6ESM	−9.962	--	−10.550	−10.440	−7.765	--	--	−8.194
TNF	1TNF	--	−9.125	−9.211	−9.112	--	−9.087	−9.178	--
	2AZ5	--	−8.046	−8.935	−8.750	--	−8.347	−8.490	--
	4TWT	--	−8.821	−8.720	−8.877	--	−8.264	−8.756	--
	5YOY	--	−7.999	−8.257	−8.168	--	−8.839	−9.304	--
TP53	1AIE	−6.648	--	−6.484	−6.514	--	--	--	−7.749
	2OCJ	−8.338	--	−8.911	−8.716	--	--	--	−8.635
	2VUK	−7.175	--	−7.766	−7.416	--	--	--	−7.200
	5O1F	−9.106	--	−9.472	−8.103	--	--	--	−8.504
IL6	1ALU	--	--	−7.205	−7.130	--	−6.422	--	--
	4CNI	--	--	−6.683	−6.948	--	−6.675	--	--
	4NI9	--	--	−6.463	−6.386	--	−6.422	--	--
STAT3	1BG1	--	--	--	--	--	--	−8.018	--
	6NJS	--	--	--	--	--	--	−8.715	--
	6TLC	--	--	--	--	--	--	−8.821	--

Note: CTS: cryptotanshinone; EA: ellagic acid; KF: kaempferol; PF: paeoniflorin; Tan IIA: tanshinone iia. --: the active component has no corresponding target, thereby no interaction and docking score.

**Table 2:** The docking parameters and scores of hub targets with their optimal ligands.

Target	PDB ID	Ligand	Affinity (kcal/mol)	Box					
				Center_x	Center_y	Center_z	Size_x	Size_y	Size_z
MMP9	4WZV	luteolin	−11.380	1.73	2.98	25.81	126	126	126
TP53	5O1F	luteolin	−9.472	128.69	84.19	−31.11	98	90	126
TNF	5YOY	CTS	−9.304	227.18	−427.50	275.50	92	90	74
IL-6	1ALU	luteolin	−7.205	2.67	−19.93	8.84	56	52	56
STAT3	6TLC	CTS	−8.821	−0.61	29.35	47.79	126	126	126

**Table 3:** The docking parameters and scores of key active components with their optimal targets.

Ligand	Target	PDB ID	Affinity (kcal/mol)	Box					
				Center_x	Center_y	Center_z	Size_x	Size_y	Size_z
luteolin	MMP9	4WZV	−11.380	4.06	8.93	23.40	126	126	126
quercetin	MMP9	4WZV	−11.140	26.63	41.54	34.35	126	126	126
baicalein	MMP9	4WZV	−10.500	1.73	2.98	25.81	126	126	126
EA	MMP9	4WZV	−9.723	1.73	2.98	25.81	126	126	126
Tan IIA	MMP9	4WZV	−9.530	1.73	2.98	25.81	126	126	126
CTS	TNF	5YOY	−9.304	14.13	57.51	45.56	92	90	74
KF	TNF	1TNF	−9.125	227.18	−427.50	275.50	68	52	58
PF	TNF	1TNF	−9.087	4.06	8.93	23.40	68	52	58

**Fig 6:**
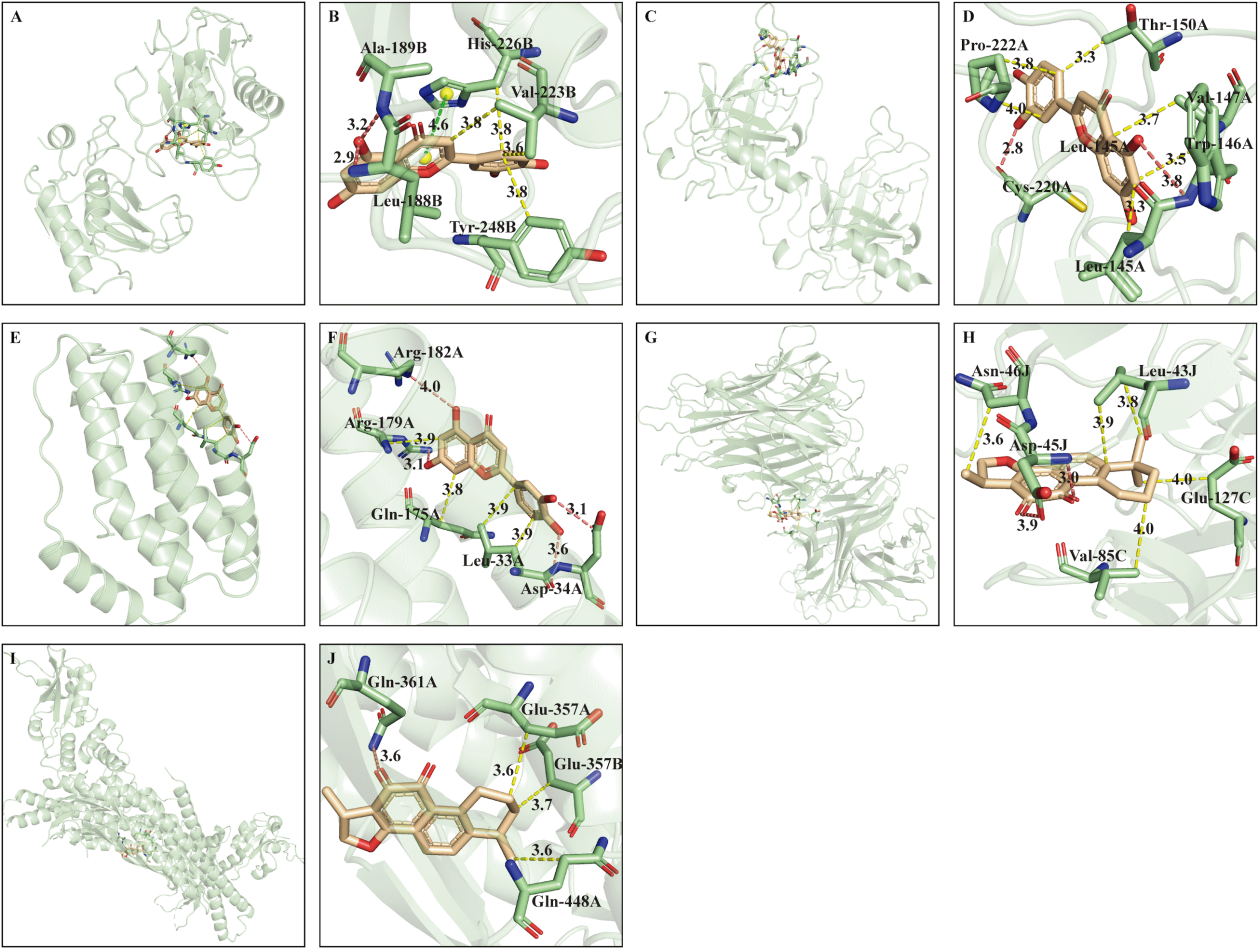
3D interaction of 5 hub targets with their optimal ligand. (A), (C) and (E) illustrated the global conformation of MMP9 (PDB ID: 4WZV), TP53 (PDB ID: 5O1F), IL-6 (PDB ID: 1ALU) interacting with luteolin; (B), (D) and (F) showed the local conformation corresponding to (A), (C), (E) in zooming mode; (G) and (I) illustrated the global conformation of TNF (PDB ID: 5YOY), STAT3 (PDB ID: 6TLC) interacting with cryptotanshinone; (H) and (J) displayed the local conformation corresponding to (G) and (I) in zooming mode. Yellow, purplish red and green dotted lines represented hydrophobic, hydrogen bond and π-stacking interactions, respectively.

**Fig 7:**
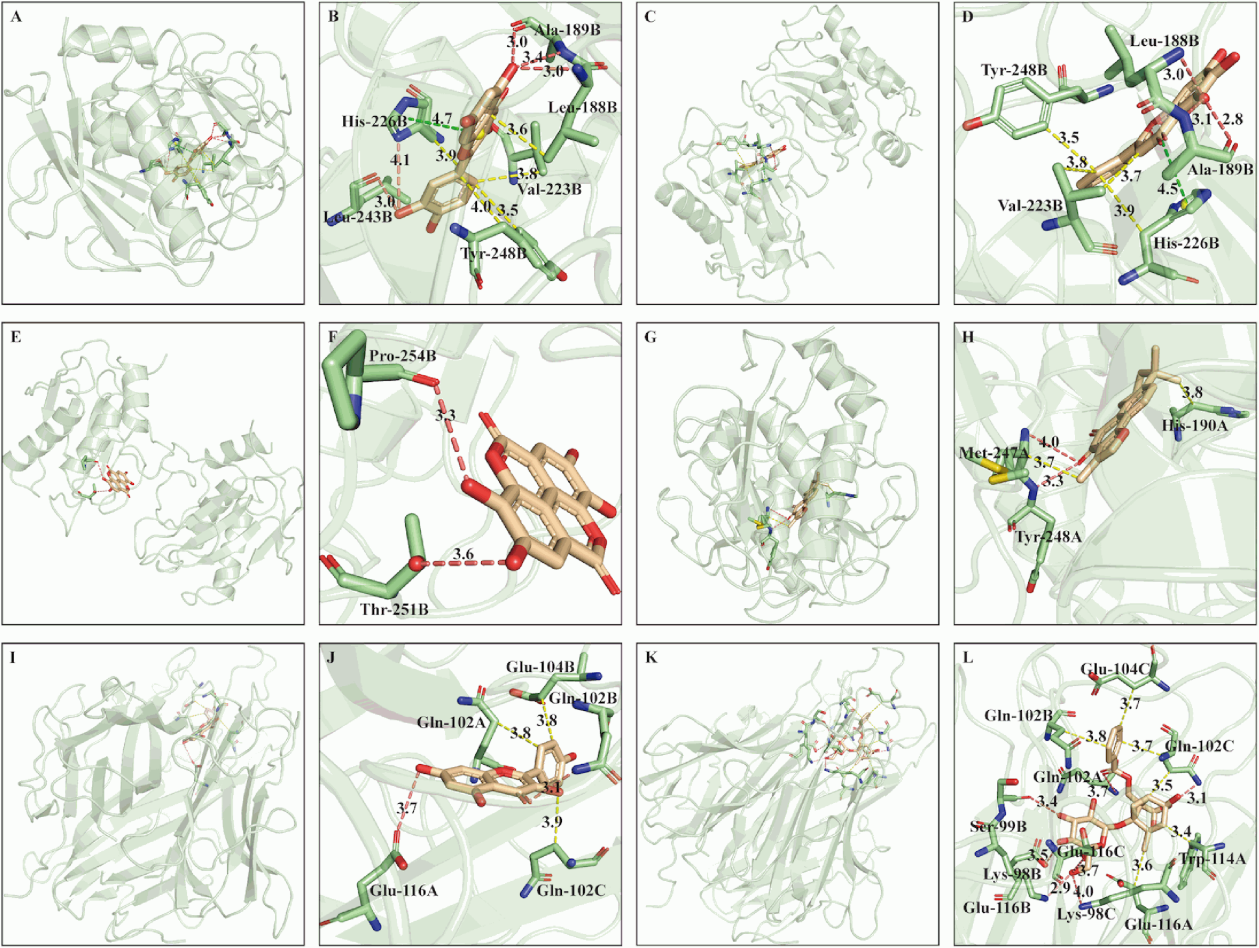
3D interaction of key active components with their optimal targets. (A), (C), (E) and (G) illustrated the global conformation of MMP9 (PDB ID: 4WZV) interacting with quercetin, baicalein, ellagic acid, tanshinone iia; (B), (D) (F) and (H) showed the local conformation corresponding to (A), (C), (E), (G) in zooming mode; (I) and (K) illustrated the global conformation of TNF (PDB ID: 1TNF) interacting with kaempferol, paeoniflorin; (J) and (L) displayed the local conformation corresponding to (I) and (K) in zooming mode. Yellow, purplish red and green dotted lines represented hydrophobic, hydrogen bond and π-stacking interactions, respectively.

For the purpose of clarifying interacting details, the complex of target and ligand with best docking conformation was utilized to profile the type and site of interaction by online tool PLIP. The 3D interaction view was visualized and displayed in whole and local zoom in manners by PyMOL. The interactions between each hub target and optimal ligand were displayed in **Fig 6** and the binding configuration of active compound-optimal target complex was illustrated in **Fig 7**. The predicted interactions primarily consisted of hydrophobic and hydrogen bonds interactions (**Figs 6**, **7**, S2–S3 Tables). Moreover, π-stacking was also observed in MMP9-luteolin, MMP9-quercetin, MMP9-baicalein complexes (**Figs 6B**, **7B**, **7D**, S4 Table).

### 3.5. XBJ inhibited LPS-induced inflammation in HEK293 cells

**Fig 8:**
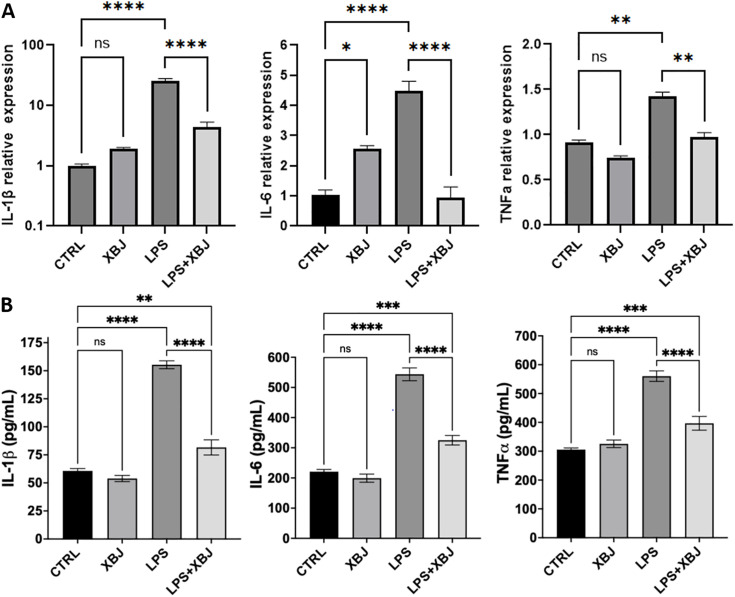
The expression of IL-1β, IL-6 and TNFα detected by qRT-PCR and ELISA in HEK-293 cells with treatment of XBJ. (A) The mRNA level of IL-1β, IL-6 and TNFα; (B) the secreted concentration of IL-1β, IL-6 and TNFα. CTRL: control; XBJ: Xuebijing (5%); LPS: lipopolysaccharides (5 µg/ml); LPS + XBJ: 5 µg/ml LPS + 5% XBJ. The data was presented as mean ± SE. *p < 0.05, **p < 0.01, ***p < 0.001, ****p < 0.0001. ns, no significance.

The network pharmacology and molecular docking analyses suggested that the protection of XBJ against sepsis and septic acute kidney injury was associated with regulation of LPS-mediated proinflammatory pathways. So, we examined whether XBJ could inhibit LPS-induced inflammation. Initially we examined potential toxicity of XBJ by using MTT assay. HEK-293 cells treated with various concentrations of XBJ showed minimal changes in viability, with all concentrations predominantly remaining at approximately 100%, without significance when comparing to the control cells (S4 Fig). This indicated the tested concentrations did not cause toxic effect and 5% of XBJ was selected as the preferred concentration for further experiments. It is well known that LPS can active the TLR4-NF-κB pathway, which leads to release of proinflammatory cytokines, such as IL-1β, IL-8, IL-18 and TNFα. Here we also found that LPS treatment resulted in significantly increased mRNA levels of IL-1β, IL-6 and TNFα, detected by qRT-PCR, when compared to the control (**Fig 8A**). Similarly, the levels of secreted IL-1β, IL-6 and TNFα, detected by ELISA, were markedly elevated, when compared to the control (**Fig 8B**). Co-treatment with XBJ led to a significant decrease in expression of IL-1β, IL-6 and TNFα at mRNA and protein levels (**Fig 8**).

**Fig 9:**
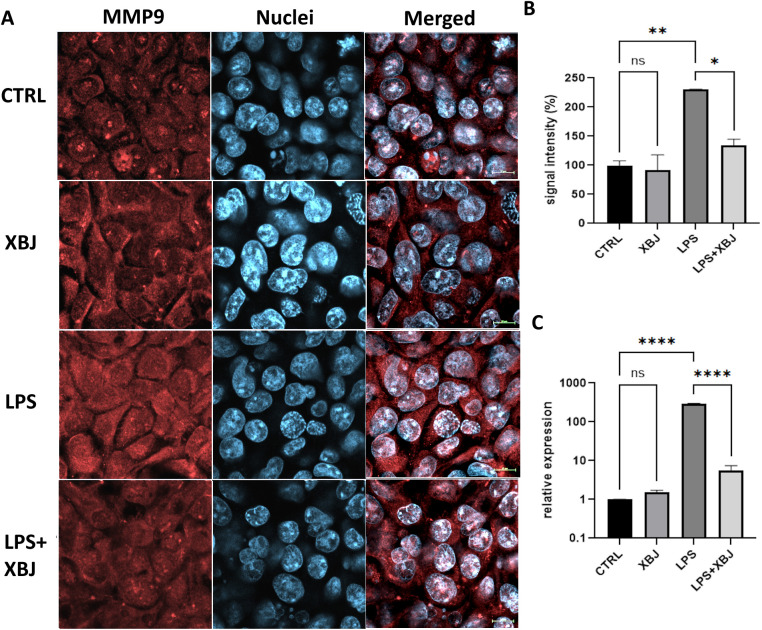
The expression of MMP9 detected by immunostaining and qRT-PCR in HEK-293 cells with treatment of XBJ. (A) Images of MMP9 immunostaining; (B) quantification of MPP9 signal intensity by using ImageJ software; (C) the mRNA level of MMP9. CTRL: control; XBJ: Xuebijing (5%); LPS: lipopolysaccharides (5 µg/ml); LPS + XBJ: 5 µg/ml LPS + 5% XBJ. The data was presented as mean ± SE. *p < 0.05, **p < 0.01, ***p < 0.001, ****p < 0.0001. ns, no significance.

MMP9 plays an important role in regulation of inflammation by activating IL-1β and cleaving some chemokines and is one of the main targets of XBJ (**Table 1**). LPS has been reported to induce MMP9 expression associated with inflammation. Here we also found that LPS treatment significantly increased MMP9 expression in HEK-293 cells by immunostaining, compared to the control. Co-treatment with XBJ significantly decreased MMP9 level, compared to cells treated with LPS alone (**Fig 9A**–**9B**). The results of qRT-PCR demonstrated that the elevated mRNA level of MMP9 induced by LPS was reversed by XBJ treatment (**Fig 9C**).

## 4. Discussion

Sepsis and related complications such as septic-AKI are leading cause of morbidity and mortality in the setting of critical illness (intensive care units) worldwide. In China, XBJ injection offers as a prevalent therapeutic strategy for sepsis and related multi-organ injury in clinical practices. However, our understanding of the molecular mechanism of XBJ treating sepsis and septic-AKI is still rudimentary. Here, we employ network pharmacology and molecular docking technologies to characterize the therapeutic targets and signaling pathways of XBJ for sepsis and septic-AKI, along with preliminary validation by *in vitro* experiments. Our results have shown that eight active ingredients (luteolin, quercetin, baicalein, ellagic acid, kaempferol, paeoniflorin, cryptotanshinone, tanshinone iia) and five hub targets (MMP9, TNF, IL-6, STAT3, TP53) might be responsible for therapeutic effect of XBJ on sepsis and septic-AKI. Among the eight active ingredients, luteolin, quercetin, kaempferol, baicalein and ellagic acid are polyphenolic secondary metabolites, ubiquitously presented in many plant taxa. Growing evidence has indicated that they possess many bioactivities, such as anti-oxidative stress, anti-inflammation, cardioprotective, hepatoprotective, nephroprotective [18–20]. Paeoniflorin is a monoterpene glycoside and the principal active constituent of Radix *Paeoniae* Alba and Radix *Paeoniae* Rubra (Chishao), which can exert protective effects on neurological, cardiovascular, and renal diseases [21]. Cryptotanshinone (CTS) and tanshinone iia (Tan IIA) are major lipid-soluble pharmacologically active constituents, belonging to abietane diterpenoids, which are isolated from *Salvia miltiorrhiza* Bunge (Danshen). CTS and Tan IIA have been proved to be effective for cardiovascular disease through improving microcirculation and enhancing vascular blood flow [22], which can also be beneficial to septic-AKI. A previous study demonstrated 90% alcohol eluent (containing over 97% total tanshiones) of Danshen extract had stronger anti-inflammatory effect in cell model, and also could significantly rescue LPS-induced septic death and improve acute kidney injury in animal model, compared to four tanshinone monomers (CTS, Tan IIA, tanshione I, dihydrotanshione) [23]. Moreover, the Danshen extract had more better pharmacokinetics parameters (C_max_, AUC) and tissue distribution than that of four tanshinone monomers [24]. These findings suggest that multiple active tanshinones play synergistic effects.

For understanding the biological function of common targets, GO and pathway enrichment analyses were performed. The identified biological processes of response to lipopolysaccharide (GO:0032496), inflammatory response (GO:0006954) were closely related to sepsis and septic-AKI. Among the five hub targets, TNF and IL-6 are proinflammatory cytokines. A systematic review and meta-analysis [25] has shown that mean concentration of TNF-α in septic patients is elevated nearly 10-fold compared to healthy individuals, and TNF-α is associated with sepsis mortality, but not sepsis severity. Recently, the work of Takahama M. et al. [26] has uncovered the pairwise effects of TNF plus IL-18, IF γ or IL-1β under the setting of sepsis, which yielded nonlinear effects on tissues through synergistic and antagonistic gene regulation. IL-6, as a major mediator of inflammation, can promote pro-inflammatory cytokine production, phagocytosis, and cell migration, such as facilitating TNF-α production via activating NF-κB pathway or canonical STAT3 pathway. MMP9 is a member of MMPs family and is also classified into the gelatinase B subgroup. MMP9 has been implicated in developmental processes, tissue remodelling, inflammatory responses and proliferative signaling pathways due to the ability of degrading extracellular matrix proteins and activating cytokines and chemokines [27]. It has been reported that MMP9 plays a bidirectional role in kidney injury [28]. In the late stages of bilateral ischaemia-reperfusion injury model, the decreased loss of renal microvessels was observed in *Mmp9*-knockout mice, which suggested that MMP9 might act as a pathogenic role in the AKI-to-CKD transition [29]. Accumulating evidence demonstrates that MMP9 induces EMT processes [30] and peritubular endothelial-to-mesenchymal transition processes (via Notch signaling) [31], as a result of promoting fibrosis. In contrast to its detrimental effects, MMP9 has the ability to degrade fibrin and reduce tubular apoptosis, which can be beneficial for AKI. Moreover, MMP9 has been proposed as an early predictive indicator for extended ICU stay following cardiac surgery with cardiopulmonary bypass [32]. Tumor suppressor p53 (TP53) is well known for its vital role in tumor suppression, cellular stress response, regulating cell cycle, apoptosis, genomic stability cell metabolism, ferroptosis, tumor microenvironment, autophagy and so on [33]. It has been reported that TP53 is an important regulator of ferroptosis [34], which functions in metabolic and immune reprogramming, inflammation and lipid peroxidation during sepsis and related acute kidney injury [35–37]. Hanna et al. [38] reveals a strong correlation between intracellular iron overload and macrophage polarization sepsis-induced kidney injury. The recent evidence has shown that immunopathology in a septic murine model is alleviated via disrupting the binding of TP53-induced glycolysis and apoptosis regulator (TIGAR) to transforming growth factor β-activated kinase (TAK1) [39]. TP53’s regulation of metabolic adaptation (e.g., TIGAR-mediated glycolysis) in immune cells [39] warrants investigation in XBJ’s mechanism. Moreover, a study demonstrates that TP53 deacetylation alleviates septic-AKI. This suggests that XBJ might exert therapeutic effect via the deacetylation of TP53, as quercetin induces activation of the deacetylase Sirtuin 1 (Sirt1). Signal transducer and activator of transcription 3 (STAT3), as a converging point of multiple inflammatory responses pathways, is a crucial modulator of sepsis [40]. STAT3 plays dual pro/anti-inflammatory roles during sepsis [40,41]. At early stage of sepsis, referred to as systemic hyperinflammatory response syndrome (SIRS), cytokines including tumor necrosis factor α (TNF‐α), interleukin (IL)‐1, IL‐2, IL‐6, are significantly elevated, which results in aberrant STAT3 activation. In turn, the activated STAT3 signaling regulates the gene expression of pro-inflammatory factors (IL-6, IFN-γ, IL-12 and IL-27), anti-inflammatory factors (IL-4, IL-10 and IL-13) and angiogenic factors (VEGF), thus forming a feedforward loop. Indeed, activated STAT3 reduces the production of TNF and IL-6 in LPS-induced septic murine model, particularly in monocytes and macrophages. Furthermore, IL-10 decreases the expression of proinflammatory IFN-induced genes via inhibiting STAT1 phosphorylation and initiates the STAT3-mediated anti-inflammatory Th2 response [41]. Accumulating evidence suggests that the cross-talking of JAK2/STAT3 (p-JAK2 and phospho-Tyr705) with other pathways such as NF-κB (p-IKKα/β, p-IκBα and p-P65), MAPK (p-JNK, pJun, p-P38 and p-ERK1/2), AKT (p-AKT), TLR4, mediate hyperinflammatory factor production during sepsis [40,42]. At the late stage of sepsis, termed as compensatory anti-inflammatory response syndrome (CARS), the persistent hyperactivation of STAT3 drives immunosuppression (“immunoparalysis”) involving diverse immune cells (Th17, Treg, myeloid-derived suppressor cells (MDSCs), macrophage), resulting in late infections and long-term mortality. Recent studies have shown that pharmacological inhibition of p-STAT3 leads to reduced proinflammatory factors, suppressed coagulation activation, improved the survival rate via inhibiting polymorphonuclear MDSCs in septic mice [42,43].

The pathway enrichment results by Metascape and STRING based on *p*-value and strength indices respectively, are not completely consistent, thereby the results can be verified with each other and be complementary. The identified common pathways are mainly related to inflammation, such as IL-10, IL-17, IL-18 signaling pathway, NF-κB signaling pathway, and the NLRP1 inflammasome. Other pathways intimately associated with sepsis and septic-AKI are also recognized in the top 10 significant pathway list, including cellular response to stress, extra-nuclear estrogen signaling, extra-cellular matrix organization, AGE/RAGE pathway, MET activating STAT3, transfer of LPS from LBP carrier to CD14. It has been reported that estrogen can improve vascular hyporeactivity in thoracic aorta induced by sepsis [44] and prevent sepsis-induced cardiac dysfunction [45]. Meanwhile, tanshinone IIA exhibits dose-dependent bioactivity mediated by estrogen receptor α and estrogen receptor β [46]. These findings support the therapeutic effect of XBJ for sepsis, at least partially. Growing evidence has shown that AGE/RAGE pathway exerts an important function in the pathogenesis of sepsis [47,48]. Besides the above pathways, the pathways enriched by STRING based on strength (calculating by log10 (observed/ expected)), describing the enrichment effect) also encompass several lipid metabolism related pathways, such as biosynthesis of electrophilic ω-3 PUFA oxo-derivative, biosynthesis of DPAn-3-derived maresins, biosynthesis of DPAn-3 SPMs, metabolism of alpha-linolenic acid (S3 Fig). Taking the pathway of biosynthesis of DPAn-3-derived maresins as an example, maresins, as novel DHA-derived lipid mediator and anti-inflammatory actors, can bring beneficial effect to sepsis and septic-AKI [49]. In sum, pathway analysis of common targets shows pertinent pathways for sepsis and septic-AKI.

To depict the interaction details between 8 active constituents and 5 hub targets, molecular docking was conducted by ADT software. Although interaction occurs in multiple ligands and targets, 8 active compounds bind to MMP9 (PDB ID: 4WZV) and TNF (PDB ID: 5YOY, 1TNF) with the lowest energy. Computational models suggest MMP9 and TNF as preferential targets of XBJ’s active constituents, but experimental validation is required. Hydrophobic and hydrogen bonds are the major interacting manners. In addition, π-stacking presented in MMP9-luteolin, MMP9-quercetin, MMP9-baicalein complexes (**Figs 6B**, **7B**, **7D**) is associated with the structure of ligand. Luteolin, quercetin and baicalein are flavonoids, whose skeleton contains A, B, C rings. The interaction of π-stacking occurs in the histidine residual (His226) on B chain of MMP9 and C ring of ligand.

The above findings suggested XBJ’s active ingredients had strong interactions with MMP9 and TNF. This was also validated by the results from *in vitro* experiments. XBJ counteracted the upregulation of IL-1β, IL-6, TNF-α and MMP-9 induced by LPS at both mRNA and protein level. However, XBJ had no significant effect on the expression of IL-1β, IL-6, TNFα and MMP-9 without LPS stimulation in HEK-239 cells. One of the potential active components of XBJ, baicalein has been reported to inhibit the expression of TNF-α, MMP-9, IL-6 in LPS-stimulated macrophages [50]. IL-6, a key inflammatory cytokine, participates in STAT3 activation and causes a significant upregulation of MMP9 [51,52]. Recently, a study reported that a natural product suppressed MMP9 expression via inhibition of the JAK/STAT3 pathway and TNFα-dependent pathways [53]. This prompted that XBJ downregulated MMP9 expression via inhibiting IL-6/JAK/STAT3 signaling axis. IL-1β, as a critical substrate of MMPs, could also induce MMP9 expression by activation of AP-1 via the JNK and ERK1/2 signaling pathways [54]. Tanshinone iia (Tan IIA), as an inhibitor of AP-1, reduced MMP-9 expression induced by IL-1β. Cryptotanshinone (CTS) also exerted an inhibitory role on MMP-9 expression [55]. As shown in the literature, TNFα stimulated MMP-9 expression, which was involved in ACSL1/JNK/ERK/NF‑kB axis [56]. Quercetin exhibited inhibitory effects on both NF-κB and AP-1 activation [57], suggesting its downregulatory effect on MMP-9. The other four potential active components of XBJ, such as kaempferol, ellagic acid, luteolin, paeoniflorin, also suppressed the expression of MMP-9 [58–60]. These lines of evidence supported our results. Thus, it was a reasonable interpretation that downregulation of IL-1β/MMP-9, IL-6/MMP-9 and TNF-α/MMP-9, at least partially, was responsible for anti-septic and anti-septic-AKI effects of XBJ. Furthermore, a large number of randomized clinical trials and related meta-analyses have demonstrated XBJ’s clinical efficacy for sepsis and other critical illnesses [13,61–63], which is closely correlated with the anti-inflammatory mechanism. Among these clinical researches, a high quality, representative example conducted by Liu et al. [63] following a multicenter, randomized, double-blind, placebo-controlled design, have shown that XBJ significantly decreased 28-day mortality in comparison to placebo, along with a favorable safety profile. The ability of XBJ reducing inflammatory factors such as C-reactive protein, IL-1β, IL-6, IL-10 and TNF-α, have been substantially indicated by lots of clinical studies [61,64,65].

### 4.1. Limitations

Although our study has predicted some potential mechanisms of XBJ against sepsis and septic-AKI, there are still two main limitations present in this study. Firstly, it is the methodological limitation in network pharmacology. This study applies conventional strategy to screen active components of herbs based on OB ≥ 30% and DL ≥ 0.18. Therefore, some potential active constituents such as hydroxysafflor yellow A (MOL002690, OB = 4.77%), oxypaeoniflorin (MOL007006, OB = 12.98%), tanshinol A (MOL007080, OB = 21.31%), hydroxysafflor yellow B (MOL002736, OB = 3.01%, DL = 0.17), protocatechuic acid (MOL000105, OB = 25.37%, DL = 0.04), danshensu (MOL007134, DL = 0.06), protocatechuic aldehyde (MOL001452, DL = 0.03) [12,66], might be omitted. Moreover, it only searches against one database (TCMSP) for obtaining XBJ’s active ingredients and targets. Secondly, this study lacks sufficient experimental validation of function (the interactions between active ingredients and hub targets) and mechanism via more proper cell models and animal models, such as cecal ligation and puncture induced septic rat model. The HEK-293 model, while useful for initial screening, cannot replicate immune-mediated sepsis pathology. Future work will validate targets in primary immune cells or animal models. Although molecular docking suggests TNF/MMP9 as primary targets, biochemical confirmation (e.g., binding assays) is essential to exclude computational artifacts. Notably, the roles of TP53 in sepsis-associated metabolic reprogramming [39] and STAT3 in immune exhaustion [41] were not explored here but represent critical pathways for future investigation of XBJ’s systemic effects.

## 5. Conclusion

**Fig 10:**
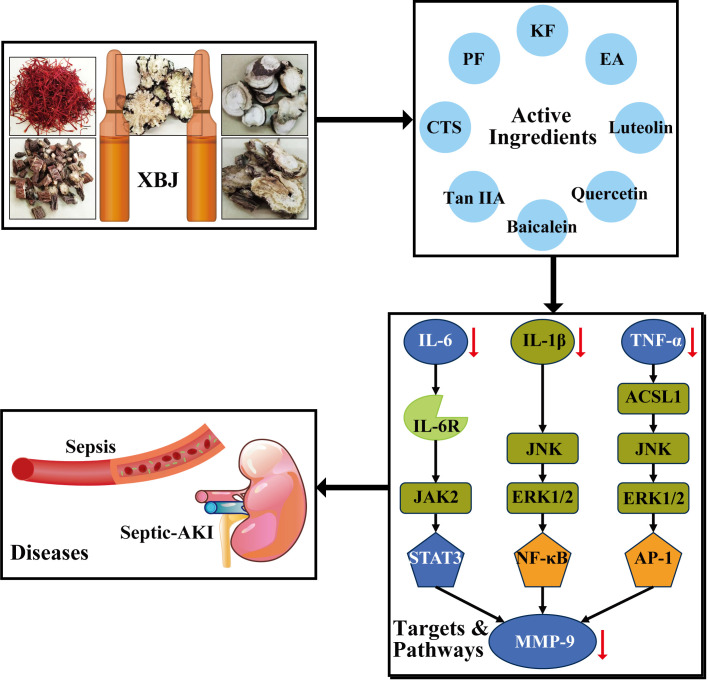
Schematic diagram of the anti-inflammation mechanism of XBJ in the treatment of sepsis and septic-AKI.

Here, we employ network pharmacology and molecular docking technologies, as well as *in vitro* experiments, to uncover the mechanism of XBJ treating sepsis and septic-AKI. Our results show that 8 active ingredients (luteolin, quercetin, kaempferol, baicalein, ellagic acid, paeoniflorin, cryptotanshinone, tanshinone iia) might exert effect through five hub targets (MMP9, TNF, IL-6, STAT3, TP53). The specific processes are mainly involved in inflammation related pathways, such as NF-κB signaling pathway, AGE/RAGE pathway, IL-10, IL-17, and IL-18 signaling pathways. Furthermore, the molecular docking results suggest that 8 active constituents have optimal docking affinity with MMP9 and TNF. The results are also initially validated by *in vitro* experiments. Collectively, as illustrated in **Fig 10**, the clinical treatment of sepsis and septic-AKI by XBJ injection has been implicated in multiple active ingredients, targets, pathways, which work synergistically and predominantly involve in anti-inflammation. The findings provide a basis for studying action of mechanism of XBJ treating sepsis and septic-AKI.

## Supporting information

S1 Fig The GO enrichment analysis of 111 common targets by STRING. (DOCX)

S2 Fig The pathway enrichment analysis of 111 common targets by STRING based on P-value. (DOCX)

S3 Fig The pathway enrichment analysis of 111 common targets by STRING based on strength. (DOCX)

S4 Fig Cell viability of HEK-293 cells after treatment with Xuebijing (from 0–5%). (DOCX)

S1 Table Primers for qRT-PCR. (DOCX)

S2 Table The predicted hydrophobic interactions by PLIP. (DOCX)

S3 Table The predicted hydrogen bond interactions by PLIP. (DOCX)

S4 Table The predicted π-stacking interactions by PLIP. (DOCX)
