# Supplementary material for: Revealing the molecular mechanisms underlying Xuebijing against sepsis and septic acute kidney injury via bioinformatics and experimental approaches
Source: PLoS One. 2025 Oct 3;20(10):e0333478. doi: 10.1371/journal.pone.0333478 (PMC12494294; doi:10.1371/journal.pone.0333478)
Supplement: S1 Fig — (DOCX) [file pone.0333478.s001.docx]

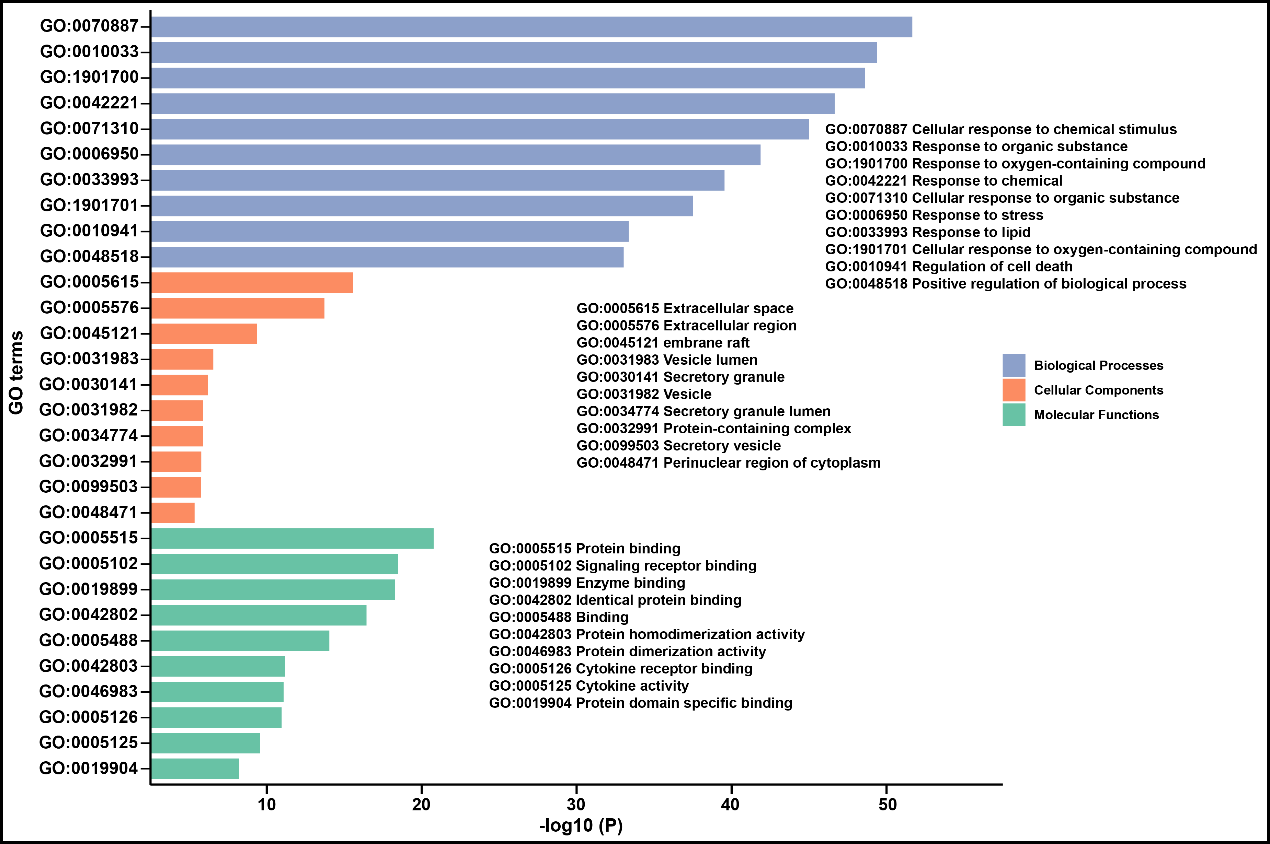


**Fig S1. The GO enrichment analysis of 111 common targets by STRING**. The GO annotation included biological processes (BP), cellular components (CC), molecular functions (MF).
