# Supplementary material for: Revealing the molecular mechanisms underlying Xuebijing against sepsis and septic acute kidney injury via bioinformatics and experimental approaches
Source: PLoS One. 2025 Oct 3;20(10):e0333478. doi: 10.1371/journal.pone.0333478 (PMC12494294; doi:10.1371/journal.pone.0333478)
Supplement: S2 Fig — (DOCX) [file pone.0333478.s002.docx]

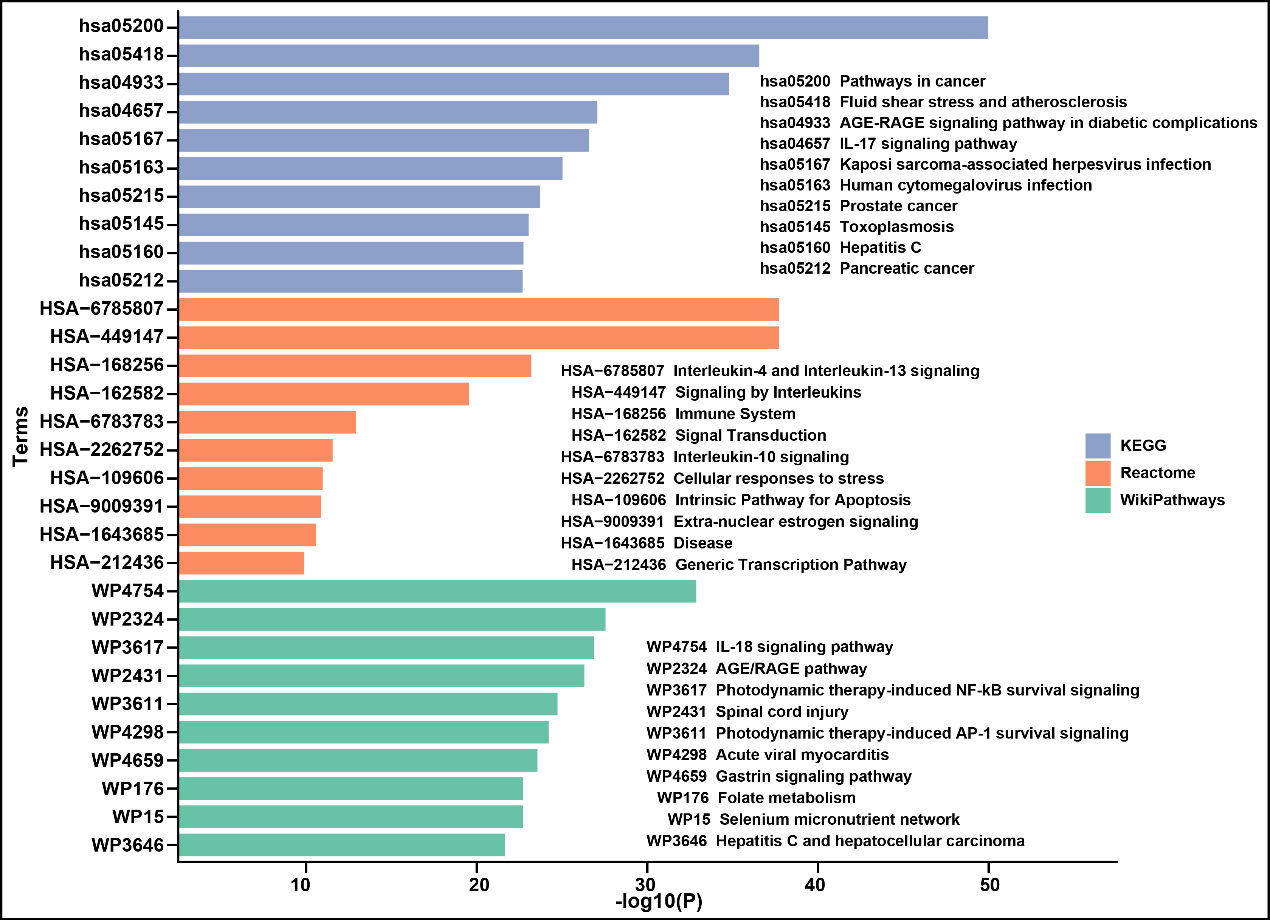


**Fig S2.** The pathway enrichment analysis of 111 common targets by STRING based on *P*-value. The pathway analysis covered three databases, including KEGG, Reactome and WikiPathways.
