# Supplementary material for: Revealing the molecular mechanisms underlying Xuebijing against sepsis and septic acute kidney injury via bioinformatics and experimental approaches
Source: PLoS One. 2025 Oct 3;20(10):e0333478. doi: 10.1371/journal.pone.0333478 (PMC12494294; doi:10.1371/journal.pone.0333478)
Supplement: S4 Fig — (DOCX) [file pone.0333478.s004.docx]

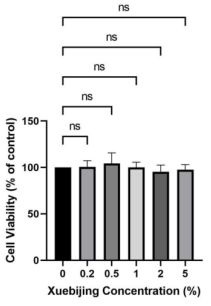


**Fig S4.** Cell viability of HEK-293 cells after treatment with Xuebijing (from 0-5%). Data was presented as mean±SE. ns: no significance.
