# Supplementary material for: Revealing the molecular mechanisms underlying Xuebijing against sepsis and septic acute kidney injury via bioinformatics and experimental approaches
Source: PLoS One. 2025 Oct 3;20(10):e0333478. doi: 10.1371/journal.pone.0333478 (PMC12494294; doi:10.1371/journal.pone.0333478)
Supplement: S1 Table — (DOCX) [file pone.0333478.s005.docx]

**Table S1** **Primers for qRT-PCR**.

| **Gene** | **Forward Primer (5’-3’)** | **Reverse Primer (5’-3’)** |
| --- | --- | --- |
| IL-1β | GCTGAGGAAGATGCTGGTTC | TCCATATCCTGTCCCTGGAG |
| IL-6 | GCA GAA AAC AAC CTG AAC CTT | ACCTCAAACTCCAAAAGACCA |
| TNFα | CCCTGAAAACAACCCTCAGA | CCACGATCAGGAAGGAGAAG |
| MMP9 | GCCACTACTGTGCCTTTGAGTC | CCCTCAGAGAATCGCCAGTACT |
| GAPDH | GAGTCAACGGATTTGGTCGT | TTGATTTTGGAGGGATCTCG |
