# Supplementary material for: Revealing the molecular mechanisms underlying Xuebijing against sepsis and septic acute kidney injury via bioinformatics and experimental approaches
Source: PLoS One. 2025 Oct 3;20(10):e0333478. doi: 10.1371/journal.pone.0333478 (PMC12494294; doi:10.1371/journal.pone.0333478)
Supplement: S2 Table — (DOCX) [file pone.0333478.s006.docx]

**Table S2 The predicted hydrophobic interactions by PLIP**.

| **Target-ligand complex** | **Index** | **Residue** | **AA** | **Distance** | **Ligand atom** | **Protein atom** |
| --- | --- | --- | --- | --- | --- | --- |
| MMP9(4WZV)-Luteolin | | | | | | |
|  | 1 | 223B | VAL | 3.8 | 17 | 2712 |
|  | 2 | 223B | VAL | 3.83 | 7 | 2711 |
|  | 3 | 226B | HIS | 3.78 | 17 | 2730 |
|  | 4 | 248B | TYR | 3.6 | 17 | 2940 |
| TP53(5O1F)-Luteolin | | | | | | |
|  | 1 | 145A | LEU | 3.28 | 11 | 490 |
|  | 2 | 147A | VAL | 3.74 | 3 | 521 |
|  | 3 | 147A | VAL | 3.48 | 11 | 520 |
|  | 4 | 150A | THR | 3.26 | 17 | 545 |
|  | 5 | 222A | PRO | 3.84 | 17 | 1272 |
|  | 6 | 222A | PRO | 3.97 | 13 | 1276 |
| TNF(5YOY)-CTS | | | | | | |
|  | 1 | 43J | LEU | 3.83 | 11 | 4807 |
|  | 2 | 43J | LEU | 3.91 | 6 | 4808 |
|  | 3 | 46J | ASN | 3.59 | 22 | 4840 |
|  | 4 | 85C | VAL | 3.97 | 8 | 3735 |
|  | 5 | 127C | GLU | 3.97 | 12 | 4135 |
| IL-6(1ALU)-Luteolin | | | | | | |
|  | 1 | 33A | LEU | 3.94 | 12 | 203 |
|  | 2 | 33A | LEU | 3.91 | 16 | 201 |
|  | 3 | 175A | GLN | 3.8 | 9 | 1490 |
|  | 4 | 179A | ARG | 3.93 | 11 | 1527 |
| STAT3(6TLC)-CTS | | | | | | |
|  | 1 | 357A | GLU | 3.68 | 8 | 2256 |
|  | 2 | 357B | GLU | 3.63 | 5 | 7551 |
|  | 3 | 448A | GLN | 3.58 | 12 | 2963 |
| MMP9(4WZV)-Quercetin | | | | | | |
|  | 1 | 188B | LEU | 3.58 | 3 | 2392 |
|  | 2 | 223B | VAL | 3.83 | 17 | 2714 |
|  | 3 | 226B | HIS | 3.86 | 17 | 2732 |
|  | 4 | 248B | TYR | 3.99 | 12 | 2940 |
|  | 5 | 248B | TYR | 3.53 | 17 | 2942 |
| MMP9(4WZV)-Baicalein | | | | | | |
|  | 1 | 223B | VAL | 3.79 | 17 | 2710 |
|  | 2 | 223B | VAL | 3.72 | 8 | 2709 |
|  | 3 | 226B | HIS | 3.86 | 17 | 2728 |
|  | 4 | 248B | TYR | 3.48 | 17 | 2938 |
| MMP9(4WZV)-Tan IIA | | | | | | |
|  | 1 | 190A | HIS | 3.78 | 11 | 797 |
|  | 2 | 247A | MET | 3.72 | 22 | 1300 |
| TNF(1TNF)-KF | | | | | | |
|  | 1 | 102A | GLN | 3.83 | 13 | 937 |
|  | 2 | 102C | GLN | 3.92 | 16 | 3830 |
|  | 3 | 104B | GLU | 3.84 | 14 | 2413 |
| TNF(1TNF)-PF | | | | | | |
|  | 1 | 102B | GLN | 3.85 | 36 | 2397 |
|  | 2 | 102C | GLN | 3.75 | 33 | 3844 |
|  | 3 | 102C | GLN | 3.46 | 5 | 3845 |
|  | 4 | 104C | GLU | 3.72 | 34 | 3873 |
|  | 5 | 114A | TRP | 3.4 | 10 | 1062 |
|  | 6 | 116A | GLU | 3.62 | 11 | 1093 |

Note: CTS: cryptotanshinone; EA: ellagic acid; KF: kaempferol; PF: paeoniflorin; Tan IIA: tanshinone iia.
