# Supplementary material for: Revealing the molecular mechanisms underlying Xuebijing against sepsis and septic acute kidney injury via bioinformatics and experimental approaches
Source: PLoS One. 2025 Oct 3;20(10):e0333478. doi: 10.1371/journal.pone.0333478 (PMC12494294; doi:10.1371/journal.pone.0333478)
Supplement: S3 Table — (DOCX) [file pone.0333478.s007.docx]

**Table S3 The predicted hydrogen bond interactions by PLIP**.

| **Target-ligand complex** | **Index** | **Residue** | **AA** | **Distance H-A** | **Distance D-A** | **Donor Angle** | **Protein donor?** | **Side chain** | **Donor Atom** | **Acceptor Atom** |
| --- | --- | --- | --- | --- | --- | --- | --- | --- | --- | --- |
| MMP9(4WZV)-Luteolin | | | | | | | | | | |
|  | 1 | 188B | LEU | 1.95 | 2.92 | 159.08 | Yes | No | 2384 [Nam] | 22 [O3] |
|  | 2 | 189B | ALA | 2.24 | 3.2 | 154.65 | Yes | No | 2393 [Nam] | 22 [O3] |
| TP53(5O1F)-Luteolin | | | | | | | | | | |
|  | 1 | 146A | TRP | 3.1 | 3.79 | 126.3 | Yes | No | 500 [N3] | 22 [O3] |
|  | 2 | 146A | TRP | 2.89 | 3.79 | 155.02 | No | No | 22 [O3] | 500 [N3] |
|  | 3 | 220A | CYS | 2.37 | 2.81 | 106.96 | No | No | 18 [O3] | 1253 [O2] |
| TNF(5YOY)-CTS | | | | | | | | | | |
|  | 1 | 45J | ASP | 2.18 | 3.04 | 141.41 | Yes | No | 4827 [Nam] | 2 [O2] |
|  | 2 | 45J | ASP | 2.93 | 3.87 | 177.35 | Yes | Yes | 4833 [O3] | 3 [O2] |
| IL-6(1ALU)-Luteolin | | | | | | | | | | |
|  | 1 | 33A | LEU | 3.16 | 3.62 | 110.47 | No | No | 20 [O3] | 200 [O2] |
|  | 2 | 34A | ASP | 2.33 | 3.06 | 130.83 | No | Yes | 18 [O3] | 212 [O.co2] |
|  | 3 | 179A | ARG | 2.76 | 3.14 | 102.29 | Yes | Yes | 1533 [Ng+] | 24 [O3] |
|  | 4 | 182A | ARG | 3.09 | 3.97 | 144.56 | Yes | Yes | 1562 [Ng+] | 22 [O3] |
| STAT3(6TLC)-CTS | | | | | | | | | | |
|  | 1 | 361A | GLN | 2.63 | 3.62 | 163.36 | Yes | Yes | 2303 [Nam] | 3 [O2] |
| MMP9(4WZV)-Quercetin | | | | | | | | | | |
|  | 1 | 188B | LEU | 1.99 | 2.98 | 164.08 | Yes | No | 2386 [Nam] | 24 [O3] |
|  | 2 | 189B | ALA | 2.43 | 3.38 | 155.69 | Yes | No | 2395 [Nam] | 24 [O3] |
|  | 3 | 189B | ALA | 2.59 | 3.05 | 109.1 | No | No | 24 [O3] | 2398 [O2] |
|  | 4 | 226B | HIS | 3.36 | 4.06 | 126.76 | Yes | Yes | 2735 [Npl] | 18 [O3] |
|  | 5 | 243B | LEU | 2.28 | 2.99 | 128.46 | No | No | 18 [O3] | 2882 [O2] |
| MMP9(4WZV)-Baicalein | | | | | | | | | | |
|  | 1 | 188B | LEU | 2.06 | 3 | 152.48 | Yes | No | 2382 [Nam] | 18 [O3] |
|  | 2 | 189B | ALA | 2.16 | 3.08 | 149.33 | Yes | No | 2391 [Nam] | 18 [O3] |
|  | 3 | 189B | ALA | 2.37 | 2.75 | 102.66 | No | No | 18 [O3] | 2394 [O2] |
| MMP9(4WZV)-EA | | | | | | | | | | |
|  | 1 | 251B | THR | 2.89 | 3.62 | 132.28 | No | Yes | 21 [O3] | 2999 [O3] |
|  | 2 | 254B | PRO | 2.83 | 3.28 | 108.67 | No | No | 19 [O3] | 3020 [O2] |
| MMP9(4WZV)-Tan IIA | | | | | | | | | | |
|  | 1 | 248A | TYR | 2.31 | 3.31 | 165.82 | Yes | No | 1316 [N3] | 3 [O2] |
| TNF(1TNF)-KF | | | | | | | | | | |
|  | 1 | 102B | GLN | 2.53 | 3.11 | 115.29 | Yes | Yes | 2386 [Nam] | 2 [O2] |
|  | 2 | 116A | GLU | 2.8 | 3.72 | 157.38 | No | Yes | 24 [O3] | 1081 [O.co2] |
| TNF(1TNF)-PF | | | | | | | | | | |
|  | 1 | 98B | LYS | 1.87 | 2.87 | 166.34 | Yes | Yes | 2366 [N3+] | 20 [O3] |
|  | 2 | 98C | LYS | 3.47 | 3.97 | 112.4 | Yes | Yes | 3813 [N3+] | 20 [O3] |
|  | 3 | 99B | SER | 2.5 | 3.44 | 161.49 | No | No | 24 [O3] | 2374 [O2] |
|  | 4 | 102A | GLN | 2.83 | 3.7 | 142.89 | Yes | No | 946 [Nam] | 31 [O2] |
|  | 5 | 102C | GLN | 2.18 | 3.06 | 144.02 | Yes | Yes | 3847 [Nam] | 2 [O3] |
|  | 6 | 116B | GLU | 2.76 | 3.49 | 132.39 | No | Yes | 22 [O3] | 2543 [O.co2] |
|  | 7 | 116C | GLU | 3.33 | 3.66 | 102.28 | No | Yes | 20 [O3] | 3989 [O3] |

Note: CTS: cryptotanshinone; EA: ellagic acid; KF: kaempferol; PF: paeoniflorin; Tan IIA: tanshinone iia.
