# Supplementary material for: Revealing the molecular mechanisms underlying Xuebijing against sepsis and septic acute kidney injury via bioinformatics and experimental approaches
Source: PLoS One. 2025 Oct 3;20(10):e0333478. doi: 10.1371/journal.pone.0333478 (PMC12494294; doi:10.1371/journal.pone.0333478)
Supplement: S4 Table — (DOCX) [file pone.0333478.s008.docx]

**Table S4 The predicted π-stacking interactions by PLIP**.

| **Target-ligand complex** | **Index** | **Residue** | **AA** | **Distance** | **Angle** | **Offset** | **Stacking Type** | **Ligand Atoms** |
| --- | --- | --- | --- | --- | --- | --- | --- | --- |
| MMP9(4WZV)-Luteolin | | | | | | | | |
|  | 1 | 226B | HIS | 4.58 | 16.15 | 1.67 | P | 1, 3, 4, 5, 6, 7 |
| MMP9(4WZV)-Quercetin | | | | | | | | |
|  | 1 | 226B | HIS | 4.67 | 26.5 | 1.62 | P | 1, 3, 4, 5, 6, 7 |
| MMP9(4WZV)-Baicalein | | | | | | | | |
|  | 1 | 226B | HIS | 4.48 | 13.62 | 1.57 | P | 1, 3, 4, 5, 7, 8 |
